# Thermophoretic tweezers for single nanoparticle manipulation

**DOI:** 10.3762/bjnano.11.97

**Published:** 2020-07-30

**Authors:** Jošt Stergar, Natan Osterman

**Affiliations:** 1 Faculty of Mathematics and Physics, University of Ljubljana, Jadranska 19, Ljubljana, Slovenia; 2 J. Stefan Institute, Jamova 39, Ljubljana, Slovenia

**Keywords:** laser, microfluidics, nano-manipulation, thermophoresis, trapping, tweezers

## Abstract

We present the trapping and manipulation of a single nano-object in an aqueous medium by optically induced temporally varying temperature gradients. By real-time object tracking and control of the position of the heating laser focus, we can precisely employ thermophoretic drift to oppose the random diffusive motion. As a result, a nano-object is confined in a micrometer-sized trap. Numerical modeling gives a quantitative prediction of the effect. Traps can be dynamically created and relocated, which we demonstrate by the controlled independent manipulation of two nanoparticles.

## Introduction

In science and technology, there is an incessant need, or at least a desire, for a (contactless) manipulation of small objects, such as nanoparticles, molecules, or even single atoms. In this work we present an approach to the thermophoretic trapping of particles in dynamic temperature gradients induced through laser heating. While it is also possible to trap atoms and molecules in vacuum using electromagnetic traps, thermal noise is a governing factor when particles are under biological conditions, namely at room temperature in an aqueous medium. Laser tweezers [1–3], invented long ago and recognized with the Nobel prize in 2018, seem like a perfect tool for such manipulations. Still, since the gradient force scales with the volume of the trapped object, only particles larger than about a few hundred nanometers in diameter can be easily trapped and manipulated in practice. A contrast in the index of refraction between the particle and the surrounding solvent is also required. For manipulation of smaller particles and molecules, typically, electrophoretic [4] and electrokinetic [5] forces are used, but they need sophisticated electrode geometries. A combination of optical tweezers and an array of nanodots, so-called plasmonic tweezers [6–8] or a fluidic slit with appropriately tailored topography with resulting spatially modulated electrostatic potential [9] can be used to trap nanoparticles, but again a prefabricated nanostructured substrate is needed.

A decade ago, the anti-Brownian electrokinetic (ABEL) trap [10–12] was invented. In the ABEL trap, the Brownian motion of a particle is optically monitored, and then a feedback electric field is applied so that the resulting electrokinetic forces induce a drift that exactly cancels the Brownian motion. This can also be achieved by moving the surrounding fluid via electroosmosis where an applied feedback electric field moves a layer of surface ions, which subsequently pulls the fluid, along with any suspended objects, by viscous drag. In such a manner, quantum dots in a liquid have been manipulated with nanometer precision [13].

Real-time force feedback can also be implemented with optical tweezers [14–16]. Recently, systems based on high-precision position detection and feedback control running at 100 kHz have been employed to generate arbitrary potentials for micrometer-sized particles [17–18].

A less commonly used principle for micromanipulation is thermophoresis [19–21], where particles or molecules are moved by a thermal gradient. Strong thermal gradients can be generated by laser heating, bringing the flexibility and microscale definition of a contact-less all-optical approach [22–23]. A combination of thermophoresis and fluid flow can be used to highly concentrate (trap) nanoparticles and molecules [24–25]. Suspended biological cells can be easily thermophoretically manipulated by harnessing the permittivity gradient in the electric double layer of the charged surface of the cell membrane [26]. Optical heating of a thermoplasmonic substrate causes a spatial separation of dissolved ions, generating a light-directed thermoelectric field, which allows for the manipulation of metal nanoparticles [27].

Recently Braun et al. [28–30] combined optical feedback and thermophoresis to create a “thermophoretic microbeaker” to confine the motion of a single nano-object. For the creation of high thermal gradients, their approach requires prefabricated plasmonic structures, which results in a big drawback: the confinement of an object is possible just within the structure, i.e., the object can not be freely manipulated everywhere in the experimental chamber.

**Figure 1:**
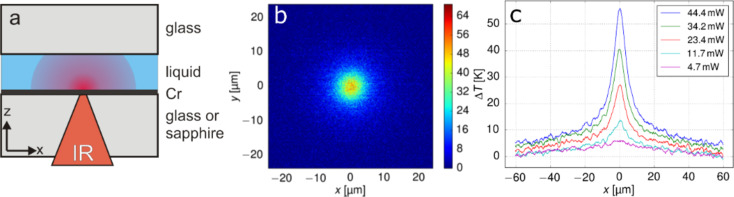
Experimental chamber and laser-induced heating. (a) Chamber cross section. A 5 μm thick liquid layer is sandwiched between a chromium-coated glass or sapphire substrate and a top coverslip. A focused IR laser beam is absorbed in the Cr layer, thus creating a hot spot. (b) Fluid temperature field for *P*_las_ = 44 mW and (c) temperature profiles through the center of the hot spot for different laser powers on a sapphire substrate.

Here we demonstrate simpler yet useful thermophoretic tweezers for nano-objects, which enables both the independent manipulation of multiple nano-objects and the creation of arbitrary trapping potentials. It is based on feedback-controlled local heating of the sample (as shown in Figure 1). The feedback loop comprises two steps. Firstly, the position of the trapped object is acquired by video-microscopy, and secondly, the heating laser is focused to such a position that the induced thermal gradient in the sample pushes the object towards the desired center of the trap. The simplicity of the design enables mostly software-based modification of an existing optical tweezers system. i.e., the video feedback loop has to be modified and a sample cell has to be constructed with an appropriate absorbing material on the substrate. Further experimental details are given in the Experimental section at the end of this paper.

Similarly to other optothermal trap designs [31–32], the approach is free of any prior modifications of the substrate such as electrodes, microchannels, biochemical or local inhomogeneous surface modifications and can thus be dynamically created in parallel and relocated to an arbitrary position. The use of multiple thermo-optical traps enables a host of exciting applications, most prominently single-molecule chemistry.

We demonstrate the thermophoretic tweezers by the trapping and parallel manipulation of individual particles with sizes of 1 μm and 200 nm in an aqueous medium. We model the effect with an overdamped Langevin dynamics simulation to obtain quantitative predictions. Since the feedback control algorithm can be easily modified, the resulting particle trapping potential can be thus arbitrarily shaped. We show this feature by creating a rectangular shaped potential well for a 200 nm nanoparticle. As a hallmark application of the thermophoretic tweezers, we demonstrate the simultaneous manipulation of two individual nanoparticles.

## Results and Discussion

### Trapping

To trap a freely diffusing nanoparticle of radius *a*, diffusion coefficient *D* = *k*_B_*T*/6πη*a*, and thermodiffusion coefficient *D_T_* = *S_T_D* (here *S_T_* is the Soret coefficient) in a solvent of viscosity η we dynamically created high-temperature gradients. To limit diffusion of the particle, one has to create an appropriate temperature gradient ∇*T* to produce a thermophoretic drift *v* = −*D_T_*∇*T* of the particle towards the desired position, i.e., to always oppose the displacement caused by random thermal positional fluctuations. Due to the continuous thermophoretic “kicks” towards the desired location, the particle is effectively trapped in a quasi-static potential.

The trapping potential was characterized using the standard procedure for optical tweezers calibration [33]: from the recorded trajectory of the particle positions, the probability density ρ(*x*) distribution was obtained. Although the discussed process is a non-equilibrium process one can still define the effective trapping potential as *U*(*x*) = −*k*_B_*T*_eff_lnρ(*x*), where *k*_B_ is the Boltzmann constant and *T*_eff_ the average effective temperature.

**Figure 2:**
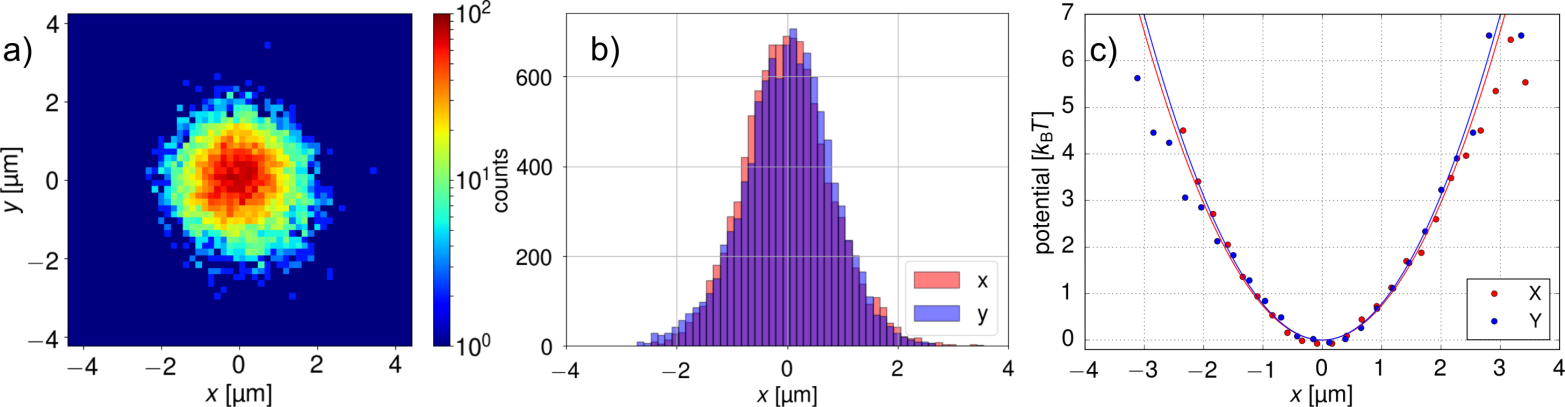
Thermo-optical trapping of a 200 nm polystyrene particle in water (*P*_las_ = 44 mW). (a) 2D histogram of particle positions. (b) Histogram of *x*- and *y*-positions. (c) Corresponding trap potentials in *x*- and *y*-directions obtained from (b), fitted with parabolic functions.

We have trapped colloidal particles of different sizes to experimentally demonstrate the flexibility and the trapping efficiency of the thermophoretic tweezers. In Figure 2, we analyze the distribution of the recorded particle positions and the corresponding effective trapping potential for 200 nm diameter polystyrene beads in aqueous 1 mM TRIS solution on a sapphire substrate. (A real-time video is available as Supporting Information File 1). It can be seen that the potential is symmetric in *x*- and *y*-directions. With the aim of comparing the trap stiffness with that of optical tweezers we have introduced a parabolic approximation of the potential around the minimum. It is important to note, however, that true form of the potential is not harmonic, but rather approaches constant force potential at larger distances, which is due to the feedback loop methodology. From the fit of parabolic function *U*(*x*) = *kx*^2^ we obtained the trap stiffness coefficient *k* = 1.47*k*_B_*T*/µm^2^ = 6.0 fN/µm. Larger particles are even easier to trap due to their lower diffusion and higher Soret coefficient. For 1 μm diameter polystyrene beads in the same solution (*D* = 0.5 µm^2^/s and *S_T_* = 10/K) we obtained *k* = 15*k*_B_*T*/µm^2^ = 61.5 fN/µm, which is a value approximately 2 to 3 orders of magnitude lower than the stiffness of typical optical tweezers experiments.

The stiffness of a parabolic trapping potential can be also determined using the equipartition theorem from the averaged square of the displacement 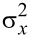 of the particle from the trap center

[1] $$k=\frac{k_{\text{B}}T}{\sigma_{x}^{2}},$$

which gives a similar result of *k* = 5.6 fN/µm for 200 nm beads.

The main difference between the “thermophoretic microbeaker” [28–29] and our thermophoretic tweezers is that the former uses a plasmonic nanostructure to create large local temperature gradients of the order of 100 K/μm, while the temperature gradients in our experiments, using a uniform substrate, are approximately ten times lower. To understand the effect of the gradient ∇*T*, one can define the characteristic trapping length from the equilibrium probability density in a temperature field [24], ρ(*x*) = ρ_0_exp(−*S_T_*Δ*T*(*x*)) = ρ_0_exp(−*S_T_*∇*Tx*) as 
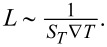
 A lower temperature gradient therefore implies a less tight trap.

### Simulation

It is clear that the trap stiffness depends on many factors, i.e., the diffusion constant of the trapped particle, the frequency of the feedback loop, and the strength of thermophoretic “kicks”, which are proportional to *D_T_* of the particle and ∇*T* at its current location. To study the effect of the factors mentioned above on the trap stiffness, we performed a simple overdamped Langevin dynamics simulation of a spherical particle in water. The laser heating was modeled as an instantly imposed Gaussian-shaped temperature field, which changes its center position according to the feedback rule. To keep the model as simple as possible, the heat capacities of the substrate and the liquid, as well as the temperature-dependent water viscosity and Soret coefficient, were not taken into account. The influence of surface proximity on the diffusion constant was neglected. For a thorough analysis of feedback-based traps in general, see [34].

The simulated equation of bead motion was

[2]
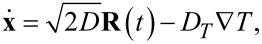


where **R**(*t*) is a delta-correlated stationary Gaussian process with zero-mean, satisfying ⟨**R**(*t*)**R**(*t*’)⟩ = δ(*t* – *t*’). The equation was solved with a finite difference method with a time step of 100 µs. The particle trajectory for a duration of 1000 s was simulated for each combination of parameters.

**Figure 3:**
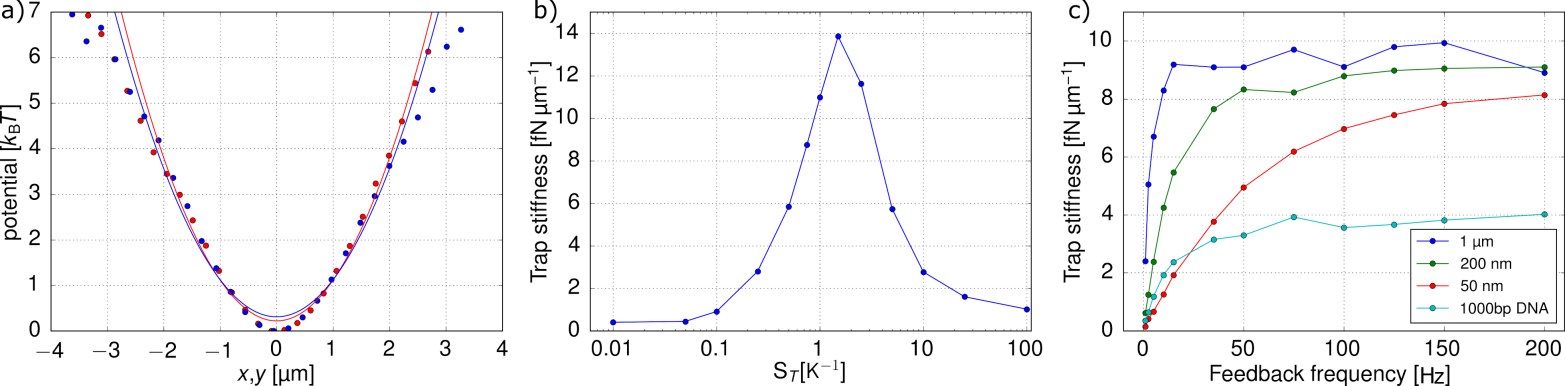
Simulation results: (a) Trapping potential in *x*- and *y*-directions, calculated from the simulated motion of a 200 nm bead. The simulation parameters are equal to the experimental conditions of Figure 2. (b) Trap stiffness at a constant feedback frequency of 35 Hz as a function of the Soret coefficient for a 200 nm bead. (c) Trap stiffness as a function of the feedback loop frequency for different particles: beads with a diameter of 1000, 200, and 50 nm (all with the same *S_T_*) and a 1000 base pairs long DNA molecule.

Figure 3 presents the simulation results. By taking parameters directly from the experiment and slightly adjusting just the full-width half-maximum of the Gaussian temperature field, which was set to 7 μm, the simulated trap potential for 200 nm particles resulted in a trap stiffness *k* = 7.2 fN/µm, which is a perfect match to the measured one, presented in Figure 2c.

The simulation also provides additional insight into the trapping details. In Figure 3b, we show the influence of the Soret coefficient on the trap stiffness for a 200 nm spherical particle, trapped by the thermophoretic tweezers, running at a constant feedback frequency of *f* = 35 Hz. At low *S_T_* values the trap is weak because the thermophoretic force is small. The stiffness grows with increasing *S_T_* up to the point where thermophoretic “kicks” towards the center of the trap are so strong that they result in an overshoot of the particle. This could be easily compensated either by lowering the heating laser power (and consequently decreasing ∇*T*) or by increasing the feedback frequency, as the average distance the particle moves during one “kick” scales with ∝*S_T_*∇*T*/*f*.

Figure 3c shows the dependence of the trap stiffness (calculated using Equation 1) on the feedback-loop frequency for three different sizes of colloidal beads with the same Soret coefficient (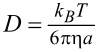, *S_T_* = 0.6) and a 1000 base pairs long DNA molecule (*D* = 8 µm^2^/s, *S_T_* = 0.3). As expected, a smaller size (i.e., larger diffusion constant) of the particles/molecules or a smaller value of *S_T_* inevitably result in a weaker trap.

At feedback frequencies lower than the characteristic particle diffusion time τ*_D_* = *a*^2^/*D* the obtained stiffness is directly proportional to the frequency, as seen in the frequency region of 0–10 Hz in Figure 3c. This can be understood from Equation 1 since the time of free diffusion, τ, before the next thermophoretic “kick” towards the trap center is inversely proportional to the feedback frequency τ = 1/*f*; therefore the expected trap stiffness is


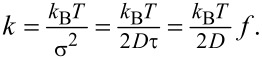


### Tailored trapping potentials

**Figure 4:**
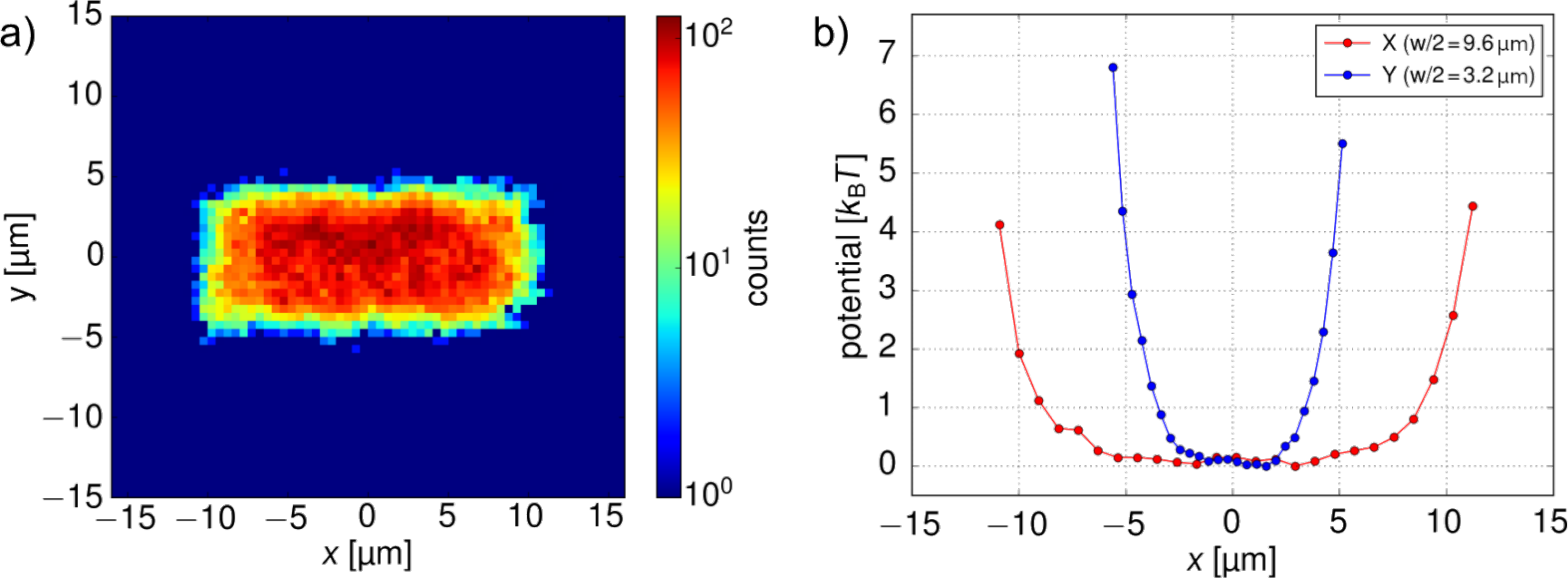
Example of a tailored nanoparticle trap. Preset feedback rules result in a creation of a 20 × 10 μm^2^ potential well for a single 200 nm particle. (a) 2D histogram of particle positions. (b) Cross section of the effective potential in *x*- and *y*-directions.

The technique is not limited to a single trapping point. Since there is no limit for the complexity of the feedback algorithm for the positioning of the hot spot, arbitrary trapping quasi-potential landscapes can be implemented. As a proof of concept, we have created a 20 × 10 μm^2^ rectangular “infinite” potential well. To achieve it, we slightly modified the feedback rule so that the force acting on the nanoparticle was always pointing towards the closest side of the rectangular region if the particle was outside the rectangle and that there was no force if the particle was inside the desired rectangle. The resulting 2D probability distribution and the potential cross sections are displayed in Figure 4. One can see the flat bottom where the particle can freely diffuse and curved edges of the potential well, which are a consequence of limited thermophoretic force acting on the particle.

### Nano-manipulation

As a hallmark application of the thermal tweezers, we demonstrate the manipulation of nanoparticles. The trapping protocol is designed so that it constantly tracks positions of the particles and corrects for possible deviations from desired locations. Particle manipulation comes naturally by merely setting the desired position away from the current particle position. In turn, a particle is steered along the line of the shortest distance to the new trapping position, achieving particle manipulation. It has to be noted, that optothermal manipulation is still a diffusive process in its core, so the resulting particle movement shows a characteristic diffusive behavior with deviations from the desired path. (The typical motion of a manipulated nanoparticle and the hot spot is presented in Supporting Information File 2).

**Figure 5:**
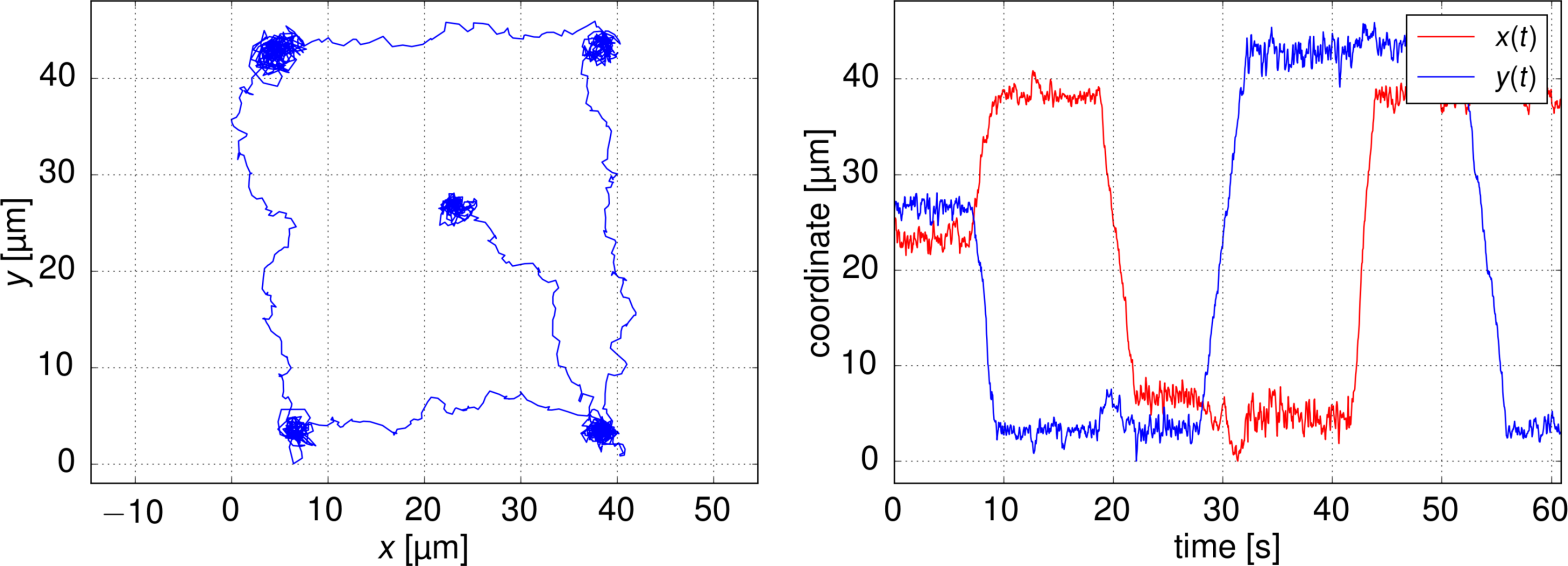
Manipulation of a 200 nm nanoparticle in water. (a) The trajectory of the particle. (b) Time dependence of *x*- and *y*-position of the particle during the manipulation.

We demonstrated successful manipulation of 1 μm and 200 nm particles (as shown in Figure 5) with average speeds of around 5 μm/s. Particles can be manipulated on two different length scales. First, the fine position of the particle inside the microscope field-of-view (FOV) can be achieved by trapping the particle and then moving the desired trap center. Larger movement exceeding the FOV can be easily reached by translating the sample mount in the microscope while the particle remains trapped in a static trap.

**Figure 6:**
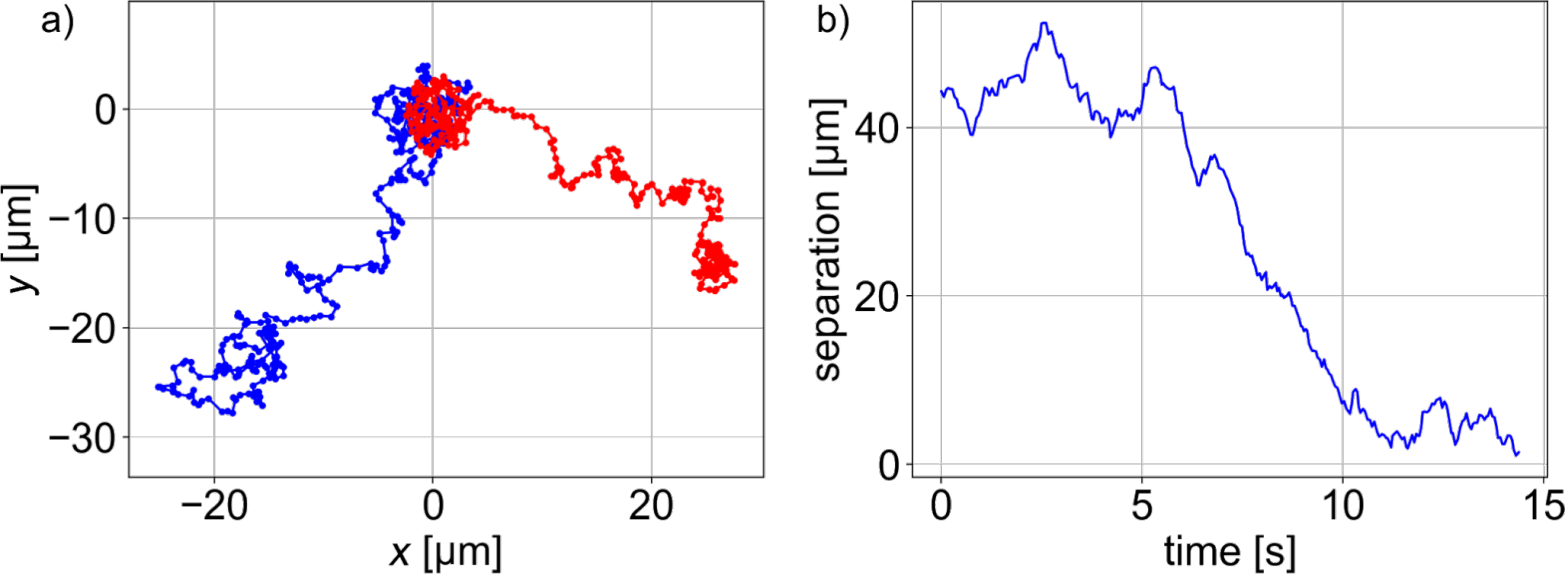
Independent manipulation of two 200 nm particles in water. (a) Particle trajectories. (b) Time dependence of interparticle separation.

As another application, we demonstrated trapping and subsequent independent manipulation of 200 nm particles. Two 200 nm particles have been trapped at a separation of 40 μm and gathered in a common final point in about 5 s, as shown in Figure 6. (A real-time video is available as Supporting Information File 3).

It can be easily imagined that instead of the nanoparticles also two fluorescently labeled molecules could be manipulated. Braun et al. [30] demonstrated the trapping of two λ-DNA molecules inside a specially designed thermophoretic trap structure with a 10–15 μm diameter. In contrast, our system enables the independent steering of particles/molecules on arbitrary paths and could be thus used as a tool for single-molecule chemistry. For example, one could imagine an experimental chamber with a few randomly diffusing labeled (bio)molecules that would never react due to the low contact probability. However, using thermophoretic tweezers, they could be easily trapped and brought close to each other to increase the reaction probability by confining them to a smaller volume.

For such single-molecule chemistry reactions, tight confinement of molecules in the “reactor” area is needed. A combination of a uniform absorbing substrate for large-scale manipulation and a central, smaller plasmonic nanostructure with a large thermal gradient, therefore, seems ideal.

One might argue about another limitation of the setup for single-molecule chemistry. The trapped molecules in the thin chamber used in our setup are always close to the surface as the vertical thermal gradient is pushing them away from the absorbing chromium layer. The molecules reach the surface at least as often as they meet each other, which might lead to a competition between the desired single-molecule chemistry and the chemistry of the molecules at the surface. This could be easily mitigated by the use of laser wavelengths with high absorption in water, such as 1.45 or 1.94 μm. Thus, the Cr layer would not be needed anymore, and the vertical thermal gradient would be much smaller than the lateral one required for trapping.

## Conclusion

We have presented a way to trap and steer nanoparticles under biological conditions using thermophoretic tweezers. A high thermal gradient, generated by the absorption of a focused laser beam, results in a thermophoretic force, typically directed away from the hot spot. By employing a feedback mechanism, which can dynamically relocate the position of the hot spot, it is possible to oppose random thermal fluctuations and therefore limit the Brownian motion of the particle.

As experimentally and numerically demonstrated, thermophoretic tweezers can be used to individually trap nano- and microparticles or molecules in a liquid environment as long as their thermodiffusion coefficient is non-zero and their spatial position can be detected. The stiffness of the trap depends on the Soret coefficient of the trapped particle, thermal gradient, and the feedback loop frequency. The numerical simulation reproduced the experimental findings very well and can be thus utilized for the estimation of the stiffness for any combination of parameters.

The all-optical creation of the tweezers allows for their dynamic relocation. Consequently, it enables the manipulation of trapped objects, which could be, for example, used to enormously increase the reaction rate of single-molecule chemical reactions.

Given the usability for research on the nanometer-scale and relative ease of implementation on existing optical tweezers systems, we anticipate that the thermophoretic tweezers could be widely used as a complementary tool to optical tweezers in biochemical, biological, and colloids research.

## Experimental

By stacking an ordinary 0.15 mm thick glass cover slide on top of a 1 mm thick substrate (sapphire window or a standard glass microscope slide) coated on the sample side with a 300 nm chromium layer by vertical deposition, a 5 μm thick experimental chamber is created (Figure 1a). All glass surfaces were thoroughly cleaned before use and treated with plasma to remove any unwanted surface contaminants. All surfaces were additionally coated with an aqueous solution of bovine serum albumin (Sigma-Aldrich) and washed to prevent nanoparticle adhesion to the glass. A collimated IR laser beam (Compass 1064-2500MN, Coherent, λ = 1064 nm) is deflected with a pair of acousto-optic deflectors (AODs). AODs enable the simultaneous generation of multiple heating spots by time-sharing (switching frequency 100 kHz) of the beam.

Behind the AODs, the laser beam is directed by mirrors and an afocal system towards a custom-built fluorescence microscope. Finally, it is focused with a long working distance IR objective (Mitutoyo M Plan Apo NIR, 50×, NA=0.42). The focused beam passes through the substrate and is absorbed in the chromium layer, thus creating a circular heated spot on the sample (*R* = 10 μm), which is up to 60 K warmer than the ambient temperature. The resulting temperature gradients reach up to 10 K/μm in the lateral direction (Figure 1b,c). Temperature measurements are performed using the temperature-dependent fluorescence of sulforhodamine B (Radiant dyes Chemie), which is calibrated in an independent measurement (accuracy ±2 K). Since the sapphire glass with a high thermal conductivity helps cooling the thin sample film, the measured characteristic cool-down time of the heated region is shorter than 20 ms, which is the typical trap relocation feedback time in the experiment. The calculated thermal relaxation time for the substrate, τ = *L*^2^/*D*_thermal_, where *L* = 10 µm and 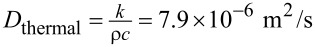 for sapphire is of the order of 10 μs.

Sample imaging is implemented using a custom-built fluorescence microscope. By using an LED-optimized filter cube (Dapi/FITC/Cy3/Cy5 Quad LED HC Filter Set, AHF analysentechnik AG), Köhler illumination is utilized for fluorescence imaging using an LED as its light source. Light is collected from the sample plane by a 63× objective (Zeiss N-Achroplan 63x/0.9W) and focused by a tube lens forming an image that is captured by a CMOS camera (BlackFly2, PointGrey Technologies).

The control feedback loop is implemented on a PC running Matlab. The loop frequency is 35 Hz. In each repetition, the image is first thresholded by quadrants to isolate fluorescent particles in the frame while eliminating possible background light. Areas of appropriate size are selected on the image, discarding too big or too small objects, corresponding to salt-and-pepper noise and aggregated blobs of particles, respectively. Centroids of remaining blobs are then calculated, thus detecting positions of the objects in the camera image. Based on the detected positions of the particles and their previous positions obtained by particle tracking, a new location of the heating spot is calculated (the spot is placed 5 μm from the current particle position so that the thermal gradient at the location of the particle is the highest). The position is then sent to the beam steering electronics, which then repositions the heating laser focus accordingly.

The most straightforward path to thermophoretic tweezing is to employ laser tweezers, which are nowadays widely used, add an optical trap repositioning feedback loop to its control software, and use a thin absorptive layer on a substrate of an experimental chamber as a heat source.

## Supporting Information

File 1 Real-time video of a trapped 200 nm polystyrene bead in water. Feedback frequency of 35 Hz. Field of view: 80 × 80 μm^2^.

File 2 Real-time video of independent manipulation of two 200 nm polystyrene beads in water. Field of view: 80 × 80 μm^2^.

File 3 Comparison of free diffusion and manipulated movement of 200 nm bead in water. Real-time video is reconstructed from the recorded particle trajectories. The red dot denotes the desired particle position; the green dot is the position of the heating laser focus, whereas the blue line represents the particle trajectory.
